# Effects of temperature and humidity on hospitalizations for metabolic syndrome with cerebral infarction among older adults in Panzhihua: a distributed lag non-linear model analysis

**DOI:** 10.3389/fpubh.2026.1674020

**Published:** 2026-03-03

**Authors:** Bingli Chen, Chunyan Zhou, Xiaoyi Liu, Deyun Luo, Jinxin Mo, Shiyang Li, Qian Zhu, Li Yin

**Affiliations:** 1 Department of Geriatrics, Panzhihua Central Hospital, Panzhihua, Sichuan, China; 2 Clinical Medical Research Center, Meteorological Medical Research Center, Panzhihua Central Hospital, Panzhihua, Sichuan, China

**Keywords:** metabolic syndrome, cerebral infarction, daily average temperature, relative humidity, distributed lag non-linear model

## Abstract

**Background:**

Research on special climatic regions, such as dry-hot valleys and high-altitude areas, is gradually emerging. Taking the dry-hot valley climate of Panzhihua as an example, this study explores the potential relationship between the risk of hospitalization due to metabolic syndrome (MetS) combined with cerebral infarction in the local older population and meteorological factors, specifically temperature and humidity.

**Methods:**

Daily meteorological data, air pollution data, and records of hospital admissions for MetS complicated with cerebral infarction at Panzhihua Central Hospital were collected from 2016 to 2020. A distributed lag nonlinear model was applied to analyze the impact of daily mean temperature and relative humidity on admission risk among adults aged over 60.

**Results:**

High temperature was associated with a reduced risk of hospital admission for MetS with cerebral infarction among older adults. The relative risk (RR) reached its minimum at lag day 17 (RR = 0.940, 95% CI: 0.887–0.996). Similarly, relatively high humidity and high humidity also reduced admission risk, with the lowest RR values observed at lag days 19 and 18, respectively. Subgroup analysis revealed that men experienced reduced admission risk when exposed to high temperature and high humidity, whereas women showed reduced risk under low temperature conditions. In the 60–75 age group, protective effects were observed with exposure to relatively high temperature, low humidity, relatively high humidity, and high humidity. However, no statistically significant effects of temperature or humidity exposure were found among individuals over 75 years of age.

**Conclusion:**

High temperature and high humidity may reduce the overall risk of hospital admission for MetS with cerebral infarction among older adults. However, these effects vary across different subgroups. Therefore, public health policies should be tailored to specific demographic groups.

## 1 Introduction

Metabolic Syndrome (MetS) is a clinical syndrome characterized by the clustering and interaction of multiple metabolic risk factors, primarily encompassing metabolic abnormalities such as obesity, hyperglycemia, hypertension, dyslipidemia, and insulin resistance (1). Substantial research has confirmed that MetS and its constituent elements serve as critical pathogenic risk factors for ischemic stroke (cerebral infarction) (2, 3). Data from population cohort studies demonstrate that compared with individuals without MetS, patients with MetS have a relative risk (RR) of developing ischemic stroke that is more than doubled (4). A meta-analysis incorporating 16 prospective cohort studies (involving a cumulative 116,496 participants) has indicated that the risk of stroke in the MetS population is significantly higher than that in the non—MetS population (5). Of note, the age at which MetS status changes is associated with the risk of stroke, and older adults are more susceptible to MetS (6). Therefore, paying attention to the population with MetS complicated by cerebral infarction is a key link in addressing the challenges of aging and public health, and identifying its risk factors is the core target for achieving simultaneous management of the two diseases.

The World Health Organization has explicitly stated that climate change has become one of the primary threats to global public health (7). As a crucial environmental variable affecting the incidence of MetS and cerebral infarction, climate change is also a core factor leading to seasonal prevalence patterns and regional distribution differences of these diseases (8–10). Temperature and humidity exhibit significant threshold effects and cumulative lag effects on the two diseases—both high and low temperature exposures may exacerbate disease progression and deteriorate prognoses (11–14). However, as a key risk factor for cerebral infarction, MetS, when combined with cerebral infarction, makes the condition more complex and the prognosis worse. Most current studies only explore the relationship between meteorological factors and MetS or cerebral infarction in isolation (15), and research on the comorbidity of the two is extremely scarce. Meanwhile, as a typical river-valley city in southwest China, Panzhihua has distinct dry-hot valley climate characteristics, with an average annual temperature of 20.3°C, annual sunshine hours exceeding 2,700 h, and a diurnal temperature range of 10–15°C (16). This unique combination of high temperature, intense sunshine, and low humidity contrasts sharply with the climate conditions of plains and coastal areas commonly studied in traditional meteorological and disease-related research. Currently, most related studies focus on temperate and subtropical climate zones, and there is almost a gap in special research on the relationship between dry-hot valley climate and health, especially MetS and cerebral infarction.

Considering the potential time-lag effects and non-linear relationships among temperature, humidity, and diseases (17, 18), this study collected daily meteorological data, air pollutant data, and hospital admission records of patients diagnosed with MetS complicated with cerebral infarction from Panzhihua Central Hospital, covering the period from 2016 to 2020. The Distributed Lag Nonlinear Model (DLNM) (19, 20), a widely used statistical tool for evaluating the associations between environmental factors and health, was employed to characterize the complex impacts of daily average temperature (DAT) and relative humidity (RH) on hospitalization rates among this patient population. This analysis aims to provide novel insights into disease incidence and progression patterns under unique climatic conditions.

## 2 Materials and methods

### 2.1 Data collection

**Figure 1:**
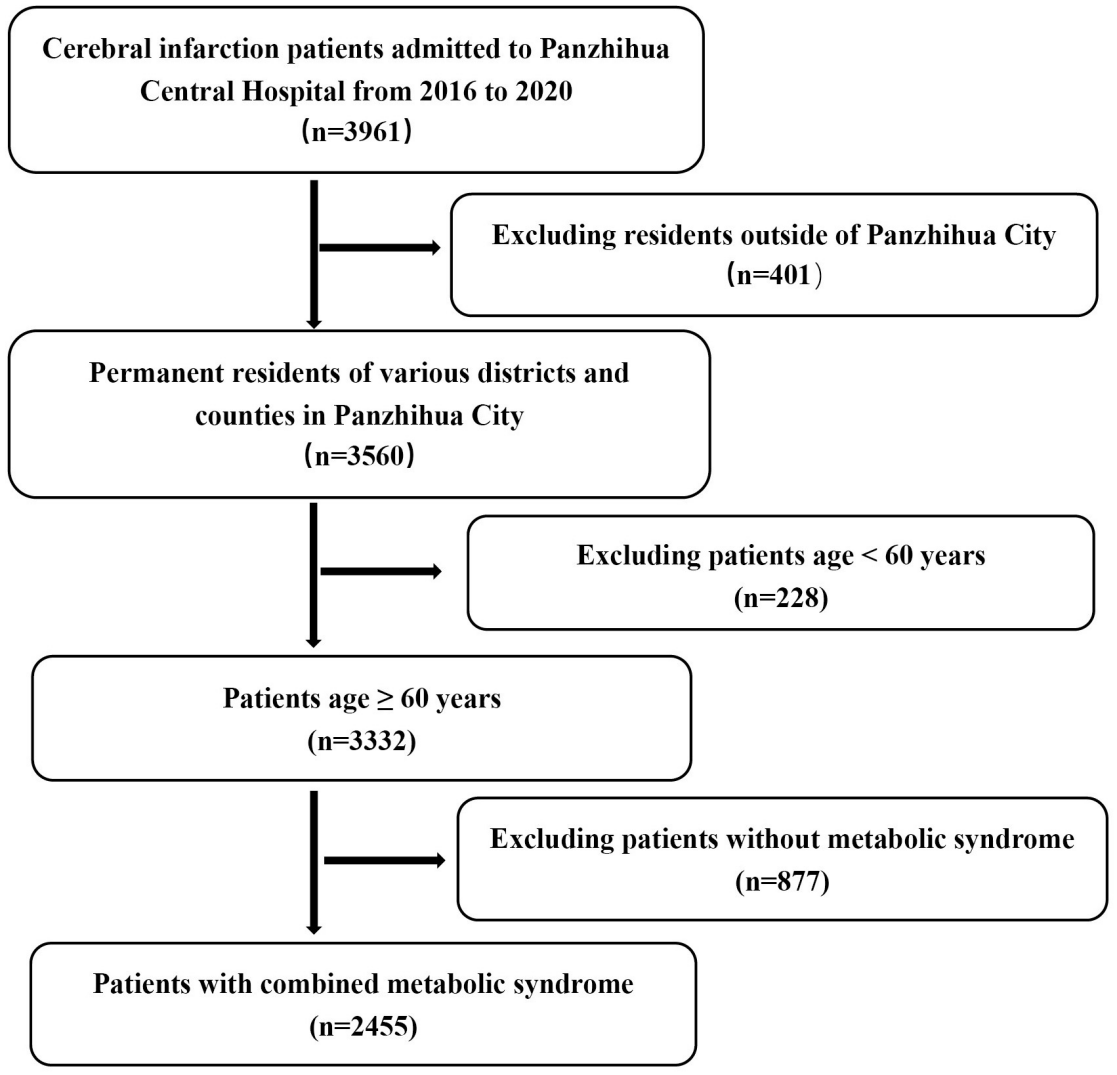
Flowchart of study population selection from Panzhihua Central Hospital database (2016-2020).

**Figure 2:**
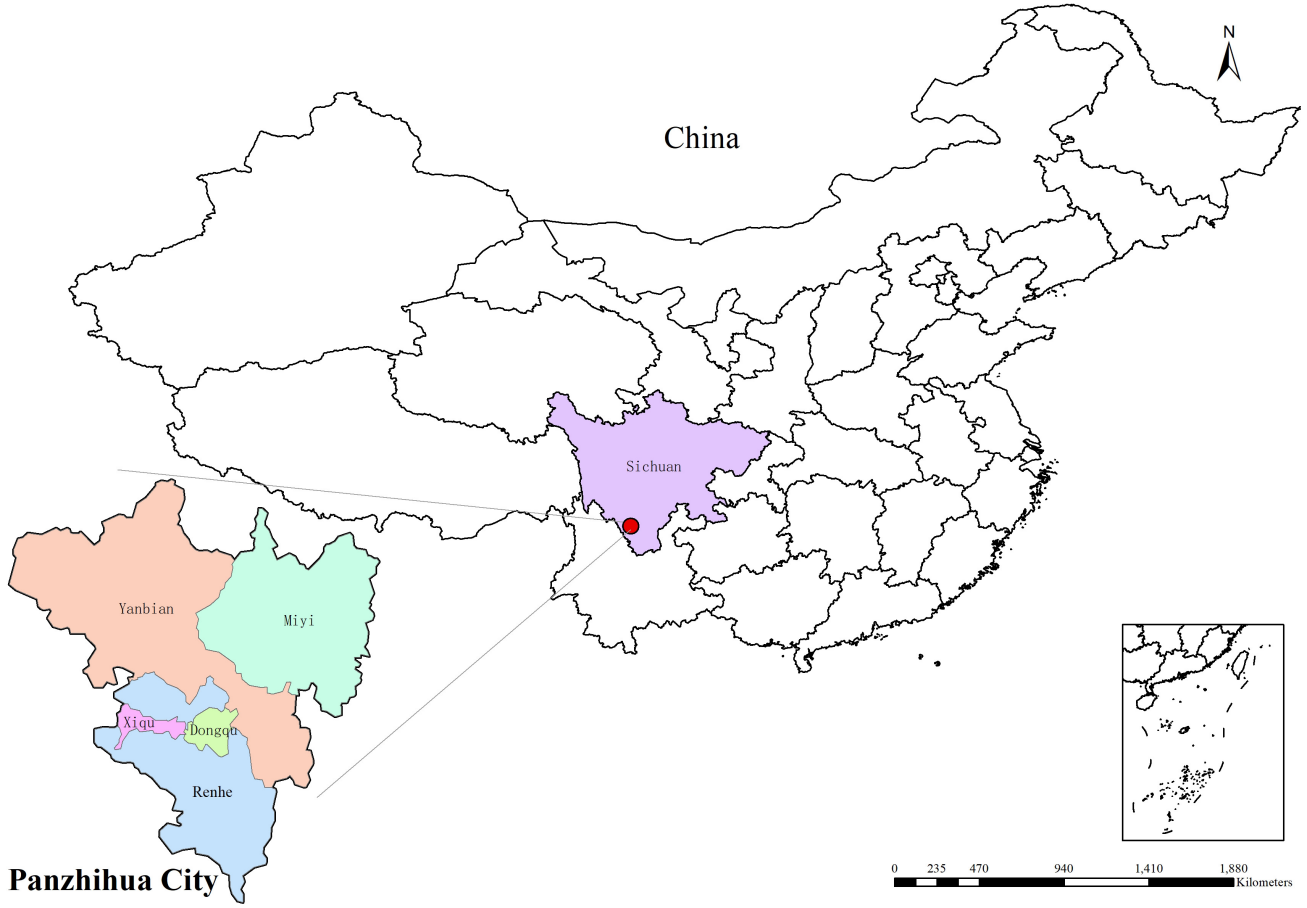
The geographical location and jurisdiction of Panzhihua city. Maps are derived from the National Platform for Common GeoSpatial Information Services (https://oauth.tianditu.gov.cn/), map approval number: GS (2024) 0650, Ministry of Natural Resources of the People's Republic of China. They were then produced using ArcMap 10.2 software.

A total of 2,455 hospital admission records for older patients with MetS complicated by cerebral infarction were retrieved from the case database of Panzhihua Central Hospital between January 2016 and December 2020 (Figure 1). These patients resided respectively in the East District, West District, Renhe District, Miyi County, and Yanbian County of Panzhihua City (Figure 2). Collected key data included admission dates, disease diagnoses, gender, age, and residence. Inclusion criteria for the study was as followed: Age ≥60 years, all patients met the diagnostic criteria specified in the Chinese Guidelines for the Diagnosis and Treatment of Acute Ischemic Stroke (2023) (21) formulated by the Chinese Neurological Association and Chinese Stroke Association, and were confirmed with cerebral infarction via cranial CT or MRI. The clinical criteria for MetS were defined according to the Guideline for the Prevention and Treatment of Type 2 Diabetes Mellitus in China (2020 edition) (22), requiring fulfillment of three or more of the following abnormalities:

(1) Abdominal obesity: waist circumference≥ 90 cm in males and≥ 85 cm in females.
(2) Hyperglycemia: fasting blood glucose ≥ 6.1 mmol/L or blood glucose ≥ 7.8 mmol/L 2h after glucose load and (or) diagnosed with diabetes and treated.
(3) Hypertension: Patients with blood pressure ≥ 130/85 mmHg (1 mmHg = 0.133 kPa) and/or confirmed hypertension who have been treated.
(4) Fasting triglycerides ≥1.70 mmol/L.
(5) Fasting high-density lipoprotein cholesterol < 1.04 mmol/L.

The daily meteorological and air pollution data for this study were obtained from the China Meteorological Science Data Sharing Service Network (http://data.cma.cn). The meteorological data included DAT, RH, daily average atmospheric pressure (DAAP), wind speed (WS), and precipitation (PRCP). The air pollutant data comprised concentrations of particulate matter ≤ 2.5 μm in aerodynamic diameter (PM_2_._5_), particulate matter ≤ 10 μm in aerodynamic diameter (PM_10_), sulfur dioxide(SO_2_), and ozone(O_3_). All data were derived from the average values recorded by five local monitoring stations. This study was approved by the research Ethics Committee of Panzhihua Central Hospital (NO: pzhszxyyll-2022-22).

### 2.2 Statistical analysis methods

We used SPSS 23.0 to summarize the daily number of hospital admissions, meteorological factors (DAT, RH, DAAP, WS, PRCP), and air pollutants (PM_2.5_, PM_10_, SO_2_, and O_3_). For the descriptive analysis, data were characterized using the mean, standard deviation, minimum, maximum, 5th percentile (P5), 25th percentile (P25), 50th percentile (P50), 75th percentile (P75), and 95th percentile (P95). A line chart was generated to illustrate the monthly average admission rate for older patients with MetS and cerebral infarction. Spearman correlation analysis was employed to assess collinearity between variables. If the correlation coefficient between any two variables exceeded 0.7 (indicating strong correlation), one of them was selectively excluded during model fitting to mitigate potential multicollinearity issues.

A DLNM was established to analyze the effects of DAT and RH on hospital visits for older patients with MetS and cerebral infarction in the Panzhihua region. The model used a quasi-Poisson function as the link function to relate meteorological factors to the number of visits. Confounding factors with a Spearman correlation coefficient |R| < 0.7 were included in the model. The correlation between PM_2.5_ and PM_10_ is strong (*R* = 0.930), and only PM_2.5_ was included in the model for analysis. The daily number of visits for MetS with cerebral infarction among older adults was treated as the dependent variable. A cross-basis was constructed for either DAT or RH and their lag periods. Other meteorological factors (WS, PRCP, and DAAP), pollutants, a natural cubic spline function of long-term trends, day-of-the-week effects, and holiday effects were incorporated into the model. The final model is expressed as follows:


$$\begin{array}{l} \text{log}\left[\text{E}\left(\text{Yt}\right)\right]=\text{α}+{\text{β}}_{1}\text{ cb}\left(\text{Tem},\text{ lag}\right)+{\text{β}}_{2}\text{ cb}\left(\text{Hum},\text{ lag}\right)\\ \;+{\text{Σ}}_{\text{NS}}\left({\text{X}}_{\text{j}},\text{ df}\right)+\text{NS}\left(\text{Pollutants},\text{ df}\right)+\text{NS}\left(\text{time},\text{ df}\right)\\ \;+\text{factor}\left(\text{DOW}\right)+\text{factor}\left(\text{Holiday}\right) \end{array}$$

Here, E (Y_t_) represents the expected daily number of hospital visits for MetS with cerebral infarction in the Panzhihua region. α is the intercept. Cb (Tem, lag) and cb (Hum, lag) represent the cross-basis matrices for DAT and RH, respectively, along with their lag dimensions. Σ NS (X_j_, df) denotes the natural cubic splines of the other meteorological confounders (WS, PRCP, and DAAP). NS (Pollutants, df) represents the natural cubic spline of the pollutant concentrations (SO_2_, O_3_, PM_2.5_). NS (time, df) is the natural cubic spline of the time variable to control for long-term trends and seasonality. DOW represents the day-of-the-week effect, and holiday represents holiday effects.

There is no definite incubation period for MetS with cerebral infarction among older adults. And this study aims to explore the acute effects of meteorological factors on diseases. Therefore, the maximum lag period is set at 21 days (23), which explores the effects of DAT and RH within 21 days of lag. According to the quasi-likelihood for Akaike's information criterion and prior research results, the degree of freedom for determining meteorological factors and their lag is 3, and the annual degrees of freedom for the time trend variable were set to 7 (24). Using the median values of temperature and humidity as references, exposure-response relationship plots illustrating the lag effects were generated. Meteorological factors were categorized into four levels: low (5th), relatively low (25th), relatively high (75th), and high (95th). Stratified analyses were conducted by gender and age to identify vulnerable subpopulations. Finally, sensitivity analyses were performed by altering the degrees of freedom for the time variable and adjusting the confounding factors included in the model.

The map of Panzhihua City was created using ArcMap 10.2 software. Data processing, statistical description, and analysis were performed using R software (version 4.3.4). The “dlnm” package was employed to establish the DLNM and to estimate the RR of DAT and RH on hospital visits for older patients with MetS complicated by cerebral infarction in the Panzhihua region. A two-tailed test was used for all statistical analyses, with the significance level set at α = 0.05.

## 3 Results

### 3.1 Descriptive summary

Table 1 describes the distribution characteristics of the daily number of older adults hospital admissions for MetS complicated with cerebral infarction, meteorological factors, and major air pollutants in Panzhihua City from 2016 to 2020. A total of 2,455 older patients were admitted for MetS with cerebral infarction. Among them, 1,409 (57.39%) were male and 1,046 (42.61%) were female. Regarding age distribution, 1,511 patients (61.55%) were aged 60–75 years, while 944 patients (38.45%) were over 75 years old. For meteorological factors in Panzhihua, the median DAT was 21.83°C (range: 5.02°C−33.55°C). The median RH was 62.60% (range: 13.90%−96.90%). The median DAAP was 878.91 hPa (range: 866.18 hPa−897.95 hPa). The median WS was 1.63 m/s (range: 0.67 m/s−4.64 m/s). The median PRCP was 0.00 mm (range: 0.00 mm−84.40 mm). Regarding air pollutants, the median concentrations were as follows: PM_2.5_ was 27.00 μg/m3 (range: 7.00 μg/m3−134.00 μg/m3), PM_10_ was 50.00 μg/m3 (range: 14.00 μg/m3−157.00 μg/m3), SO_2_ was 30.00 μg/m3 (range: 9.00 μg/m3−137.00 μg/m3), and O_3_ was 77.00 μg/m3 (range: 14.00 μg/m3−196.00 μg/m3).

**Table 1:** Admission characteristics, meteorological conditions, and pollutants of daily older patients with metabolic syndrome complicated with cerebral infarction in Panzhihua City from 2016 to 2020.

**Variables**	**Counts**	**Mean ±SD**	**Min**	**P5**	**P25**	**P50**	**P75**	**P95**	**Max**
Admissions	2,455 (100.00)	1.34 ± 1.27	0	0	0	1	2	4	8
Male	1,409 (57.39)	0.77 ± 0.90	0	0	0	1	1	2	6
Female	1,046 (42.61)	0.57 ± 0.80	0	0	0	0	1	2	5
65–75years	1,511 (61.55)	0.83 ± 0.98	0	0	0	1	1	3	7
>75years	944 (38.45)	0.52 ± 0.76	0	0	0	1	1	2	5
DAT (°C)	–	21.02 ± 5.45	5.02	12.28	16.30	21.83	25.23	29.10	33.55
RH (%)	–	58.65 ± 18.32	13.90	27.01	43.78	62.60	72.55	84.99	96.90
DAAP (hPa)	–	879.11 ± 5.05	866.18	871.20	875.55	878.91	882.35	887.85	897.95
WS (m/s)	–	1.82 ± 0.66	0.67	1.07	1.33	1.63	2.20	3.13	4.64
PRCP (mm)	–	2.43 ± 7.28	0.00	0.00	0.00	0.00	0.65	15.17	84.40
PM_2.5_ (μg/m^3^)	–	28.24 ± 11.67	7.00	13.00	20.00	27.00	34.00	49.00	134.00
PM_10_ (μg/m^3^)	–	51.88 ± 18.31	14.00	27.00	38.00	50.00	63.00	84.00	157.00
SO_2_ (μg/m^3^)	–	32.05 ± 13.29	9.00	14.00	23.00	30.00	38.00	56.00	137.00
O_3_ (μg/m^3^)	–	80.85 ± 29.95	14.00	38.00	59.00	77.00	101.00	135.00	196.00

DAT, daily average temperature; RH, relative humidity; DAAP, daily average atmospheric pressure; WS, wind speed; PRCP, precipitation; PM_2.5_, particulate matter ≤ 2.5 μm in aerodynamic diameter; PM_10_, particulate matter ≤ 10 μm in aerodynamic diameter; SO_2_, sulfur dioxide; O_3_, ozone.

Analysis of hospital admission timing for older patients with MetS complicated with cerebral infarction showed that the highest number of admissions occurred in May, while the lowest was recorded in February. A declining trend in hospital admissions was observed starting from October, followed by an increasing trend beginning in February (Supplementary Figure 1). Regarding meteorological data in Panzhihua City, the highest monthly average temperature was 27.05°C in June, and the lowest was 13.40°C in January. The lowest monthly average RH was 35.17% in March, while the highest was 77.80% in September (Supplementary Figure 2).

Spearman correlation analysis was conducted to examine the interrelationships between the meteorological factors and air pollutants. The results indicated that DAT exhibited a negative correlation with RH, DAAP, PM_2.5_, PM_10_, and SO_2_. Conversely, RH was negatively correlated with WS, PM_2.5_, PM_10_, and O_3_. DAT showed positive correlations with WS, PRCP, and O_3_, while RH was positively correlated with DAAP, PRCP, and SO_2_ (Table 2).

**Table 2:** Spearman's correlation coefficients meteorological factors and atmospheric pollutants.

**Variables**	**DAT**	**RH**	**DTR**	**DAAP**	**WS**	**PRCP**	**PM_2.5_**	**PM_10_**	**SO_2_**	**O_3_**
DAT	1									
RH	−0.076^**^	1								
DAAP	−0.669^***^	0.238^***^	−0.046	1						
WS	0.268^***^	−0.610^***^	0.083^***^	−0.395^***^	1					
PRCP	0.202^***^	0.598^***^	−0.605^***^	−0.065^**^	−0.045	1				
PM_2.5_	−0.498^***^	−0.086^***^	0.370^***^	0.270^***^	−0.280^***^	−0.395^***^	1			
PM_10_	−0.460^***^	−0.148^***^	0.423^***^	0.300^***^	−0.277^***^	−0.464^***^	0.930^***^	1		
SO_2_	−0.356^***^	0.231^***^	0.099^***^	0.276^***^	−0.393^***^	−0.051^*^	0.453^***^	0.493^***^	1	
O_3_	0.554^***^	−0.543^***^	0.318^***^	−0.504^***^	0.414^***^	−0.172^***^	−0.020	−0.049^*^	−0.284^***^	1

^*^P < 0.05.

^**^P < 0.01.

^***^P < 0.001.

### 3.2 Effects of DAT and RH on hospital admissions for older patients with MetS and cerebral infarction

**Figure 3:**
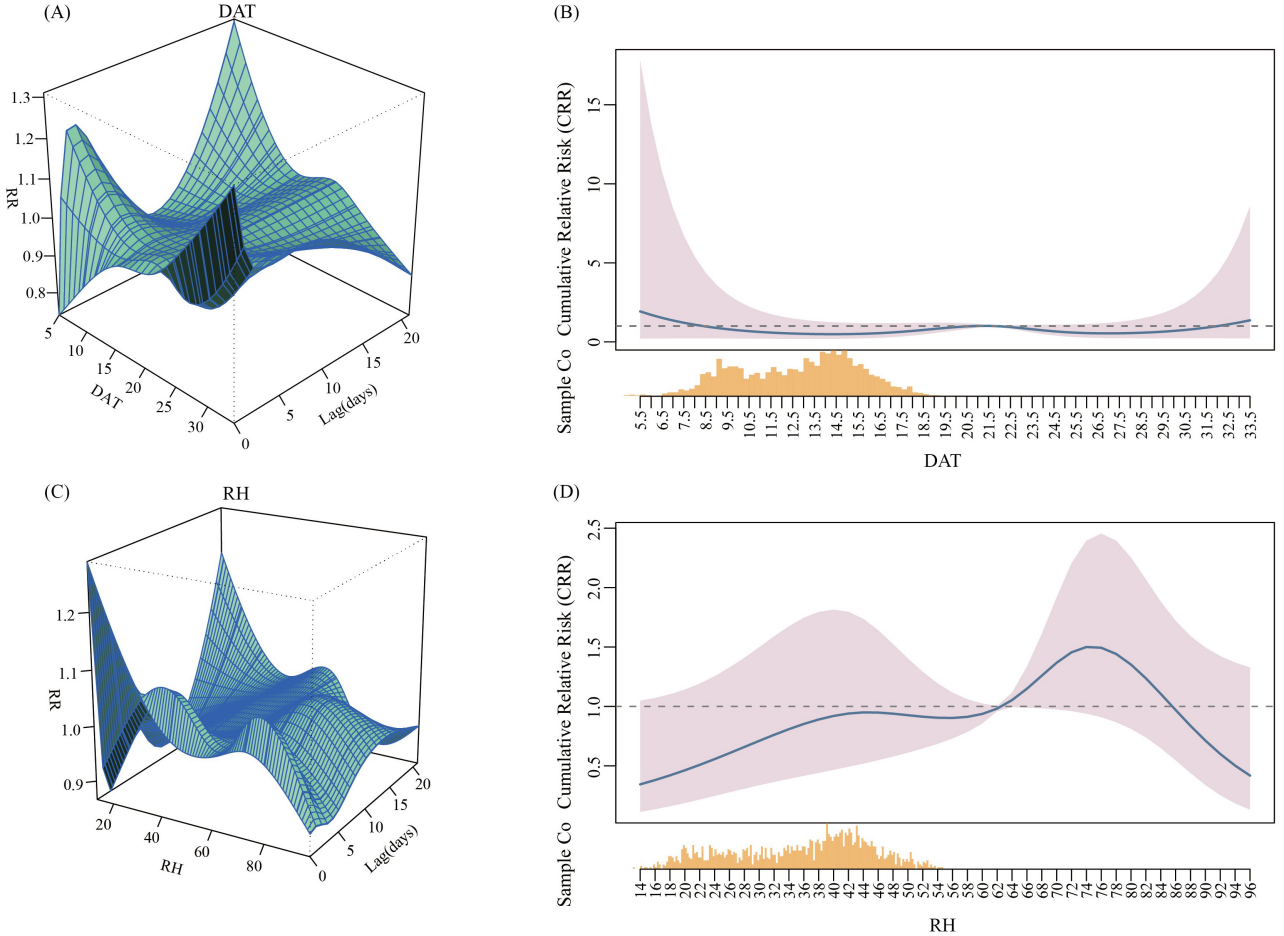
Three dimensional graph and overall exposure response correlation curve of DAT and RH on hospitalization of older patients with metabolic syndrome complicated with cerebral infarction. **(A)** The 3D graph of the relationship between DAT and hospitalization in older patients. **(B)** The overall exposure response correlation curve of the relationship between DAT and hospitalization in older patients. **(C)** The 3D graph of the relationship between RH and hospitalization in older patients. **(D)** The overall exposure response correlation curve of the relationship between RH and hospitalization in older patients.

Figure 3 displays the overall exposure-response relationships between DAT, RH, and the risk of hospital admissions for older patients with MetS and cerebral infarction across different lag days. Using the 50th percentile of mean temperature (21.8°C) and the 50th percentile of RH (62.6%) as reference values, no significant cumulative effects of DAT or RH on admissions were observed in this study. The lag-effect analysis revealed that high DAT (29.1°C) was associated with a reduced risk of hospital admissions for MetS complicated with cerebral infarction among older adults. The protective effect was strongest at lag day 17, where the RR reached its most pronounced value (RR = 0.940, 95% CI: 0.887–0.996). Subsequently, the RR began to increase, indicating a weakening of the protective effect, which disappeared entirely by lag day 20. No significant associations were observed for other DAT levels (Table 3). Regarding the lag effects of humidity, relatively high humidity (72.55%) showed a protective effect. The most significant risk reduction was observed at lag day 19 (RR = 0.947, 95% CI: 0.907–0.989), after which the RR started to increase (Table 4).

**Table 3:** The lag effect of different DAT on hospitalization treatment of older patients with MetS complicated with cerebral infarction in different lag days.

**Lag days**	**5th percentile (12.3°C)**		**25th percentile (16.3°C)**		**75th percentile (25.2°C)**		**95th percentile (29.1°C)**	
	**RR**	**95%CI**	**RR**	**95%CI**	**RR**	**95%CI**	**RR**	**95%CI**
0	0.913	0.595–1.401	0.972	0.713–1.291	1.067	0.880–1.294	1.167	0.822–1.657
1	0.973	0.806–1.175	0.976	0.859–1.109	0.956	0.873–1.045	0.992	0.853–1.155
2	1.010	0.811–1.257	0.982	0.847–1.139	0.942	0.854–1.040	0.961	0.810–1.140
3	1.023	0.904–1.157	0.988	0.907–1.075	0.975	0.920–1.034	0.996	0.903–1.098
4	1.021	0.917–1.136	0.989	0.916–1.067	0.999	0.945–1.055	1.022	0.935–1.117
5	1.008	0.899–1.131	0.986	0.909–1.070	1.009	0.951–1.069	1.034	0.940–1.137
6	0.990	0.892–1.100	0.982	0.911–1.058	1.009	0.955–1.065	1.034	0.947–1.129
7	0.970	0.891–1.057	0.976	0.918–1.039	1.003	0.958–1.049	1.028	0.957–1.105
8	0.953	0.887–1.024	0.971	0.921–1.024	0.996	0.957–1.036	1.019	0.959–1.083
9	0.941	0.877–1.009	0.967	0.918–1.019	0.989	0.952–1.028	1.010	0.952–1.072
10	0.933	0.867–1.003	0.964	0.914–1.018	0.984	0.945–1.024	1.001	0.942–1.065
11	0.928	0.860–1.002	0.962	0.610–1.017	0.979	0.939–1.020	0.992	0.931–1.058
12	0.928	0.859–1.002	0.961	0.908–1.017	0.975	0.935–1.017	0.983	0.922–1.049
13	0.930	0.863–1.002	0.960	0.909–1.015	0.972	0.933–1.012	0.975	0.915–1.038
14	0.935	0.871–1.004	0.961	0.911–1.012	0.969	0.932–1.008	0.966	0.909–1.026
15	0.943	0.882–1.007	0.961	0.915–1.010	0.967	0.932–1.003	0.957	0.904–1.014
16	0.953	0.893–1.016	0.963	0.918–1.010	0.965	0.931–1.001	0.949	0.897–1.003
17	0.964	0.902–1.031	0.964	0.918–1.014	0.964	0.928–1.000	**0.940**	**0.887–0.996**
18	0.978	0.906–1.055	0.967	0.914–1.022	0.963	0.924–1.003	**0.932**	**0.873–0.994**
19	0.992	0.906–1.087	0.969	0.907–1.035	0.962	0.917–1.009	**0.923**	**0.855–0.997**
20	1.008	0.902–1.126	0.971	0.897–1.052	0.961	0.908–1.017	0.915	0.835–1.003
21	1.024	0.896–1.170	0.974	0.886–1.071	0.960	0.898–1.027	0.907	0.813–1.011

The bold values represents statistically significant results.

**Table 4:** The lag effect of different RH on hospitalization treatment of older patients with MetS complicated with cerebral infarction at different lag dates.

**Lag days**	**5th percentile (27.01%)**		**25th percentile (43.78%)**		**75th percentile (72.55%)**		**95th percentile (84.99%)**	
	**RR**	**95%CI**	**RR**	**95%CI**	**RR**	**95%CI**	**RR**	**95%CI**
0	1.123	0.800–1.577	1.007	0.809–1.252	1.018	0.882–1.175	0.981	0.737–1.304
1	1.037	0.883–1.217	1.080	0.967–1.206	1.065	0.988–1.147	1.033	0.924–1.154
2	1.005	0.835–1.209	1.072	0.951–1.210	1.055	0.976–1.141	1.021	0.903–1.153
3	0.999	0.900–1.110	1.027	0.954–1.106	1.022	0.972–1.076	0.984	0.914–1.059
4	0.992	0.905–1.088	0.996	0.929–1.068	1.005	0.956–1.057	0.966	0.902–1.034
5	0.981	0.889–1.083	0.980	0.910–1.055	1.003	0.951–1.056	0.965	0.898–1.037
6	0.969	0.884–1.062	0.973	0.909–1.043	1.009	0.962–1.058	0.976	0.913–1.042
7	0.957	0.886–1.033	0.973	0.918–1.032	1.020	0.979–1.062	0.991	0.938–1.048
8	0.948	0.887–1.012	0.975	0.926–1.027	1.029	0.992–1.068	1.006	0.959–1.056
9	0.942	0.884–1.004	0.977	0.929–1.027	1.036	0.998–1.087	1.016	0.970–1.065
10	0.939	0.880–1.003	0.978	0.929–1.030	1.038	0.999–1.078	1.022	0.973–1.073
11	0.940	0.878–1.006	0.980	0.929–1.033	1.037	0.997–1.078	1.023	0.973–1.076
12	0.943	0.880–1.010	0.987	0.930–1.034	1.033	0.993–1.074	1.020	0.969–1.074
13	0.948	0.886–1.015	0.981	0.932–1.034	1.026	0.988–1.066	1.014	0.964–1.066
14	0.956	0.896–1.020	0.982	0.934–1.033	1.017	0.981–1.054	1.004	0.958–1.053
15	0.966	0.907–1.028	0.982	0.936–1.031	1.006	0.972–1.041	0.992	0.948–1.038
16	0.977	0.919–1.039	0.983	0.937–1.031	0.993	0.960–1.026	0.978	0.936–1.022
17	0.990	0.929–1.055	0.983	0.936–1.032	0.978	0.946–1.012	0.962	0.919–1.007
18	1.004	0.936–1.077	0.983	0.931–1.037	0.963	0.928–1.000	**0.945**	**0.898–0.994**
19	1.020	0.940–1.106	0.983	0.924–1.046	**0.947**	**0.907–0.989**	**0.927**	**0.873–0.984**
20	1.036	0.941–1.140	0.983	0.914–1.057	**0.931**	**0.884–0.980**	**0.909**	**0.846–0.976**
21	1.052	0.940–1.179	0.983	0.902–1.070	**0.915**	**0.861–0.973**	**0.890**	**0.818–0.970**

The bold values represents statistically significant results.

### 3.3 Stratified analysis by sex and age

**Figure 4:**
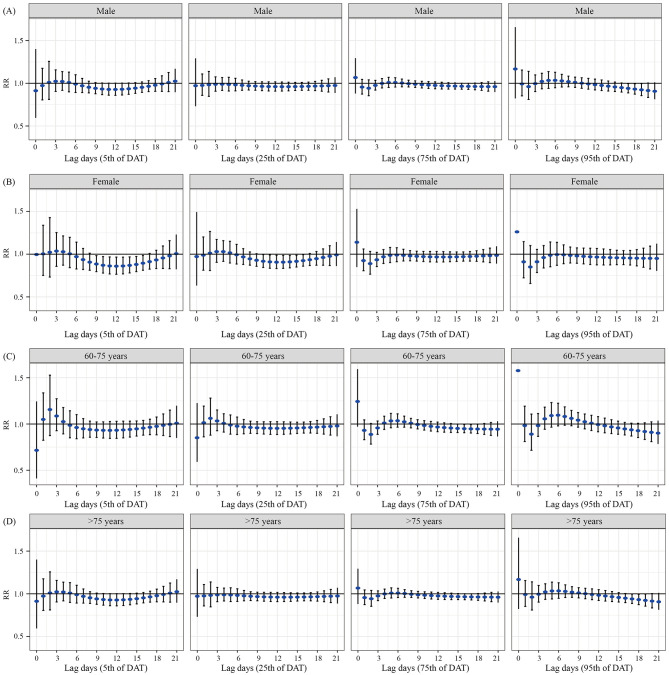
Lag effects of various DAT on hospital admissions for patients with MetS complicated with cerebral infarction, stratified by gender and age. **(A)** The lag effect of DAT on hospital admissions among older male patients. **(B)** The lag effect of DAT on hospital admissions among older female patients. **(C)** The lag effect of DAT on hospital admissions in the 60–75 years age group. **(D)** The lag effect of DAT on hospital admissions in the population aged over 75 years.

Figure 4 presents the results of the stratified analysis for DAT by sex and age. We found a protective effect when males were exposed to high temperature, with the strongest effect observed at lag day 18 (RR = 0.919, 95% CI: 0.846–0.997). This effect lost statistical significance by lag day 20. For females, exposure to low temperature was associated with a reduced admission risk, with the peak effect observed at lag day 16 (RR = 0.894, 95% CI: 0.812–0.985). Similarly, exposure to relatively low temperature also showed a protective effect in females, with the most significant risk reduction at lag day 16 (RR = 0.924, 95% CI: 0.859–0.995). In the 60–75 years age group, relatively high temperature was associated with a decreased admission risk for MetS complicated with cerebral infarction. The most pronounced effect was observed at lag day 15 (RR = 0.952, 95% CI: 0.908–0.999), after which the RR began to increase, and the association was no longer statistically significant by lag day 19. No statistically significant associations were found for temperature exposure in the population aged over 75 years.

**Figure 5:**
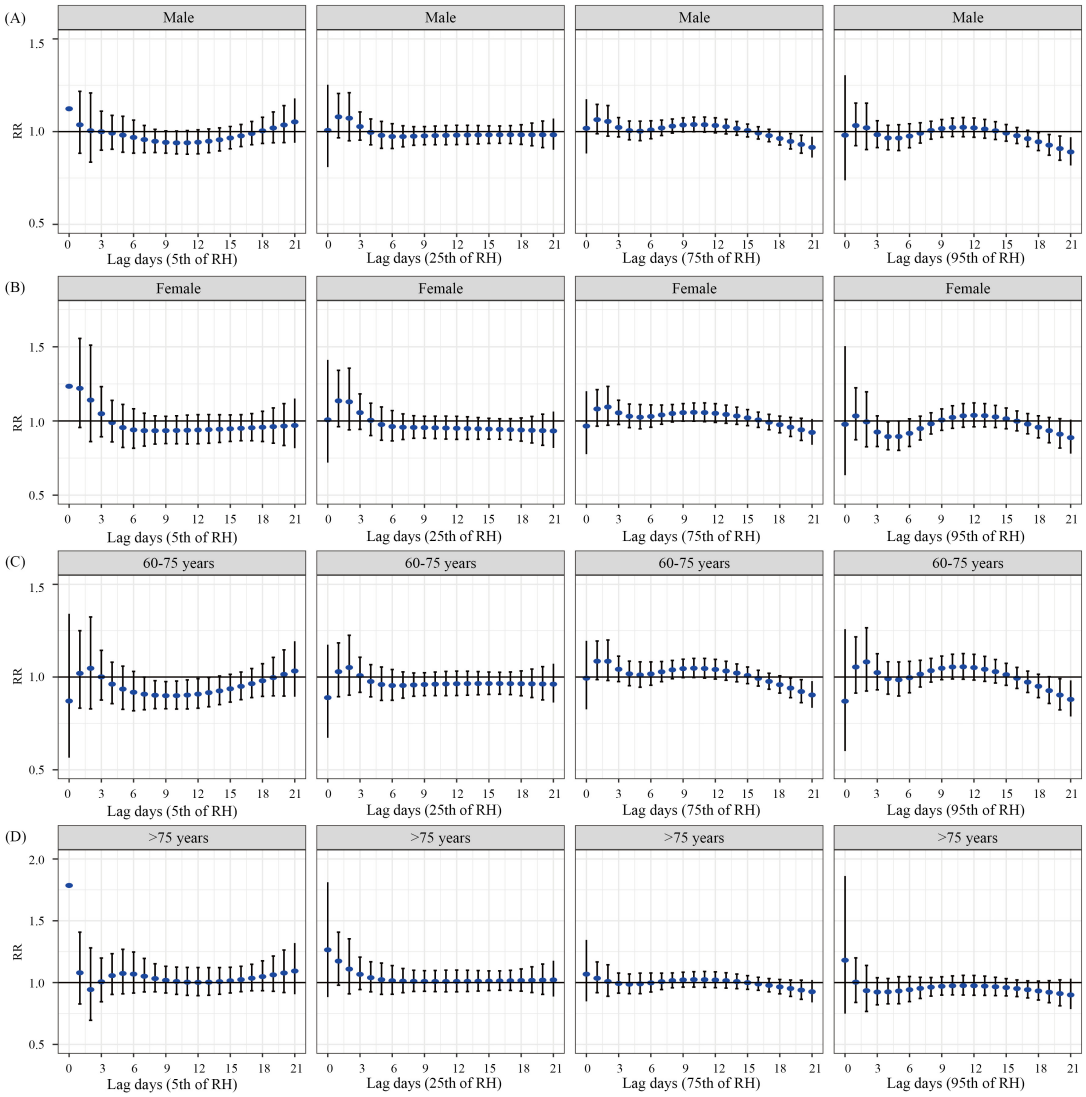
Lag effects of various RH on hospital admissions for patients with MetS complicated with cerebral infarction, stratified by gender and age. **(A)** The lag effect of RH on hospital admissions among older male patients. **(B)** The lag effect of RH on hospital admissions among older female patients. **(C)** The lag effect of RH on hospital admissions in the 60–75 years age group. **(D)** The lag effect of RH on hospital admissions in the population aged over 75 years.

The results of the stratified analysis for RH by different sex and age groups are shown in Figure 5. For males, exposure to relatively high humidity and high humidity was associated with a reduced admission risk. The strongest protective effects were observed at lag day 19 (RR = 0.947, 95% CI: 0.907–0.989) and lag day 18 (RR = 0.945, 95% CI: 0.898–0.994), respectively. In the 60–75 years age group, exposure to low humidity, relatively high humidity, and high humidity demonstrated protective effects. The most significant risk reductions were observed at lag day 13 (RR = 0.916, 95% CI: 0.840–0.998), lag day 19 (RR = 0.940, 95% CI: 0.889–0.995), and lag day 20 (RR = 0.903, 95% CI: 0.823–0.991), respectively. No statistically significant associations were found for humidity exposure in females and in the population aged over 75 years.

### 3.4 Sensitivity analysis and interaction effects

**Figure 6:**
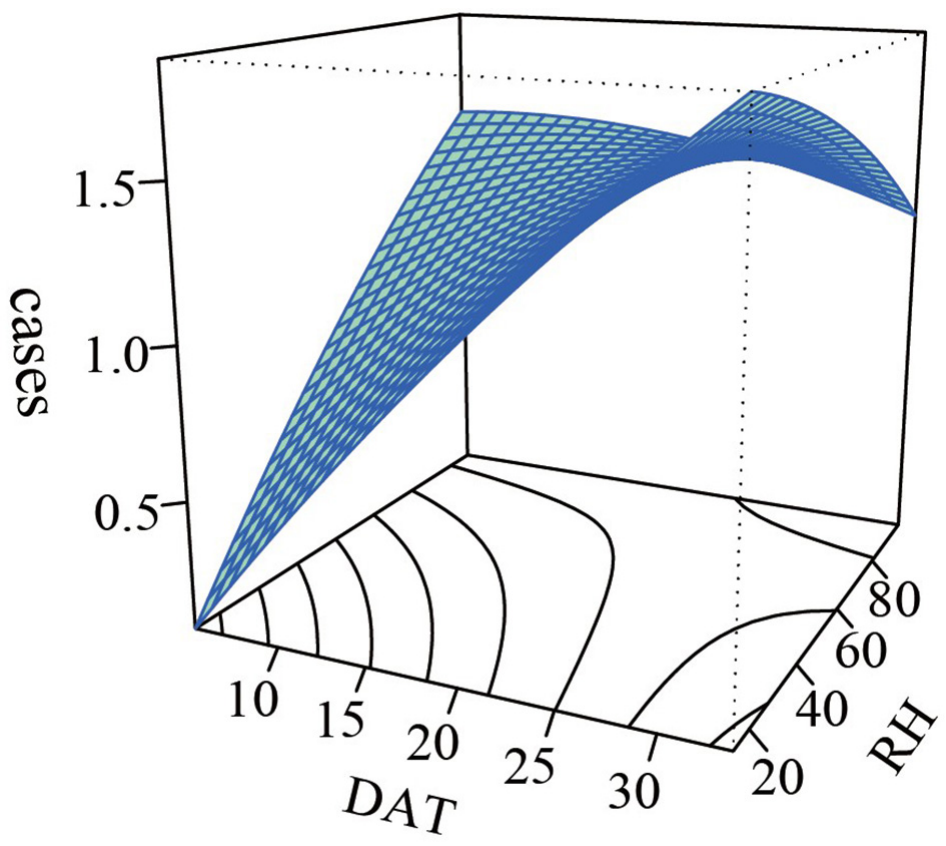
The interaction between DAT and RH on older MetS complicated with cerebral infarction.

We conducted sensitivity analyses by modifying the degrees of freedom for lag days and the confounding meteorological factors included in the model. The maximum cumulative lag effects of mean temperature and its corresponding values showed no substantial changes, indicating the robustness of the model (Supplementary Table 1). A non-parametric bivariate response surface analysis was used to model the interaction effect of DAT and RH on the incidence of MetS with cerebral infarction in the older population, as shown in Figure 6. The results suggested a potentially higher admission risk for MetS with cerebral infarction in environments characterized by high temperature and low humidity; however, the interaction effect was not statistically significant (*P* > 0.05). The interaction analysis after stratifying temperature and humidity by percentiles into three groups (P33 and P66, P25 and P75) showed no statistically significant interaction between the two factors (*P* > 0.05) (Supplementary Tables 2, 3). This indicates that variations in RH are unlikely to modify the effect of DAT on the risk of hospital admissions for MetS with cerebral infarction.

## 4 Discussion

With rapid economic development, population aging has become an increasingly serious concern. Concurrently, the impact of climate change on human health is receiving growing attention. This study provides the first comprehensive investigation and analysis of the relationship between DAT, RH, and the risk of hospital admissions for MetS complicated with cerebral infarction among the older population in Panzhihua. Exposure to high temperatures was found to reduce the risk of admission in the older population. Similarly, exposure to conditions of relatively high humidity and high humidity also lowered admission risk for these patients. These findings collectively suggest that the local climate, particularly during periods of high temperature and high humidity, may provide more suitable living conditions for older adults with MetS and cerebral infarction.

A study on stroke admissions in Guangzhou reported an approximate J-shaped relationship between temperature and hospitalizations, with low temperatures accounting for a 9.06% increase in stroke admissions (25). Another study indicated that low temperatures elevate the risk of ischemic stroke admissions among Beijing residents, demonstrating a cumulative lag effect (26). Research from Taiwan showed that lower temperatures increase the risk of type 2 diabetes, with each 1°C decrease associated with a 7% rise in HbA1c levels (27). Furthermore, existing evidence suggests that hypertension is linked to seasonal variations in ambient temperature, particularly the significant impact of low temperatures (28). Potential mechanisms derived from relevant studies suggest that low temperatures may increase sympathetic nervous system activity, promote vasoconstriction, and elevate blood pressure (29). This can lead to a higher incidence of hypertension, which is a key component of MetS, consequently influencing the risk of cardiovascular and cerebrovascular diseases. Additionally, cold exposure may lead to increased consumption of calories and fats, potentially resulting in weight gain, dyslipidemia, and hyperglycemia (30). In contrast, appropriately high temperatures may be associated with increased physical activity, greater sunlight exposure, and improved blood glucose control. These factors could subsequently contribute to a reduced prevalence of MetS and its individual components (31). This may appropriately explain that exposure to high temperatures (29.1°C) can reduce the risk of hospital admission for older patients with metabolic syndrome combined with cerebral infarction. However, some studies report contrasting findings (32, 33). Research has linked hot weather to increased risks of dehydration, elevated blood viscosity, and higher incidence of stroke, hypertension-related visits (34), and morbidity/mortality among diabetics (35). These findings contradict our results, likely due to Panzhihua's relatively mild climate where the maximum average temperature observed did not exceed 33.55°C.

Our study found that high temperatures have a protective effect on males, while low temperatures have a protective effect on females. A study from Wuhu (36) suggested that women appear to be more sensitive to high temperatures than men, showing a direct impact from extremely high mean temperatures. This is potentially because women typically have a higher body fat percentage than men, which enhances their cold tolerance but reduces their likelihood of sweating in hot environments. This, in turn, may render women less tolerant of high temperatures. Furthermore, our findings indicated that relatively high temperatures reduced admission risk for MetS complicated with cerebral infarction in the 60–75 age group. We speculate this is related to Panzhihua's dry-hot valley climate, where the generally moderate average temperatures may encourage outdoor physical activity, indirectly reducing the risk of MetS and cardiovascular and cerebrovascular incidents. Previous studies have shown that extreme temperatures, both high and low, significantly increase mortality risk among older adults (37). A study by Yang across 16 Chinese cities found that both high and low temperatures had a greater impact on stroke mortality in males and older adults (38). Another report on the environment and older adults (39) found that older adults are more sensitive to immediate exposure to high temperatures. Our study found no protective effect of temperature exposure in the population aged over 75 years. This may be associated with a diminished capacity for recovery and adaptation to temperature changes in advanced age. Research by Lin (40) noted that the body's ability to effectively control and regulate temperature declines with age.

Current findings on the relationship between MetS, cerebral infarction, and humidity remain inconsistent. Magalhães (41) reported that each 1% increase in RH was associated with a 1% higher stroke risk in northern Portugal. Similarly, a Boston study (42) indicated that cerebral infarction was linked to meteorological conditions with higher mean RH. An African study (43) found most diabetes cases occurred during the most humid months, with slightly higher admission rates for diabetic patients in the rainy season. Zhang (36) observed in a subgroup analysis that extremely high RH increased MetS mortality during hot seasons. Conversely, no correlation was found between stroke incidence and humidity variation in northern Korea (44). A Chinese study suggested that higher temperature and RH might reduce the risk of cardiovascular and cerebrovascular diseases among older adults (45). Our study demonstrated protective effects of both relatively high and high humidity against hospital admissions for older patients with MetS complicated by cerebral infarction. We speculate that the potential mechanisms for this protective effect may involve the following aspects: Firstly, environments with high humidity may help maintain the body's fluid balance and reduce the risk of dehydration, thereby exerting a protective effect on the cardiovascular and cerebrovascular systems (46). Furthermore, RH may further influence human health by affecting the concentration of air pollutants. Studies have indicated that exposure to ambient particulate matter is associated with the acceleration of atherosclerosis progression through the formation of reactive oxygen species and systemic inflammation (47). Regarding the impact of RH on particulate matter concentration, research suggests a negative correlation between RH and PM_2.5_. A possible explanation is that fine particles generated from combustion are hydrophilic; these hygroscopic particles tend to aggregate under strong intermolecular gravitational forces with water molecules, leading to an increase in the size of fine particles and facilitating their settlement (48). Additionally, high humidity exhibits an antagonistic effect with carbon monoxide, while low humidity shows a synergistic effect with carbon monoxide. Therefore, higher humidity levels may contribute to reducing air pollutants (46). This also provides a plausible explanation for why high RH exposure can lower the overall admission rate for older patients with MetS combined with cerebral infarction.

Humidity affects human health through two primary pathways: thermoregulation and water metabolism. High humidity in summer impedes the body's heat dissipation by reducing evaporative cooling through sweat, potentially leading to elevated body temperature, increased heart rate, and hypertension (49). Conversely, low RH may cause fluid loss, resulting in hemoconcentration, elevated blood pressure, and higher cerebral infarction risk (50). Existing studies report that excessive humidity correlates with higher diabetes incidence (51), while excessive dryness may increase stroke occurrence (52). Previous research also identified a positive association between RH and MetS among women, though this was not observed in men or across different age groups (53). In aging Japanese society, both lower temperatures and extremely low or high humidity were linked to increased cerebrovascular disease admissions (54). Subgroup analysis revealed a protective effect of high humidity exposure in males and adults aged 60–75, while no statistically significant association was observed for females or individuals over 75 years. We hypothesize that the attenuated effect in older adults may be attributed to increased indoor time due to declining physical function and comorbidities, potentially diminishing climate impact. These differential effects across subgroups likely reflect variations in social behaviors, self-protection awareness, and disease pathogenesis. Given the limited research on humidity's impact on older patients with MetS and cerebral infarction, further investigation is warranted to clarify these contradictory findings.

To further investigate the potential interaction between DAT and RH, we conducted non-parametric bivariate response surface analysis and tertile-based stratification analysis. However, neither analysis revealed a statistically significant interaction effect (*P* > 0.05). We speculate that possible reasons include: the weak negative correlation between DAT and RH (r = −0.076), limited confounding from multicollinearity in estimating the interaction effect, and the potential influence of sample size (55). Previous studies have found that the combination of low temperature and high humidity exerts the greatest impact on the mortality burden of cardiovascular and cerebrovascular diseases. Intriguingly, our analysis indicated that the combination of low temperature and high humidity showed a *P*- value approaching 0.05, suggesting a marginally significant protective interaction effect. Future studies with larger sample sizes are warranted to further explore this relationship.

This study employed advanced statistical methods to assess the relationship between DAT, RH, and the co-morbidity of MetS with cerebral infarction in older adults. Furthermore, data were stratified by sex and age to provide more detailed results, which aids in designing targeted interventions for susceptible subpopulations. In conjunction with a review of existing literature (Supplementary Table 4), we identified a relative scarcity of research on the influence of meteorological conditions on MetS complicated by cerebral infarction, particularly within the context of a dry-hot valley climate. This represents a novel research direction. Moving forward, researchers should develop novel detection technologies for early, rapid, and accurate identification of MetS and cerebral infarction patients. Collaboration with governments is also essential to formulate policies such as monitoring populations exposed to low DAT and RH, providing screening services, and establishing meteorological health risk warning systems. In addition, stratified and seasonal health guidelines should be developed to encourage older adults to engage in appropriate outdoor activities and ensure adequate sunlight exposure during high DAT and RH periods. Optimizing medical resource allocation, integrating these measures into chronic disease management platforms, and enhancing patients' self-management skills will enable continuous and dynamic health management.

Nevertheless, this study has several limitations. First, the focus on a single city may restrict the generalizability of findings to other climatic regions. Second, reliance on data from one tertiary hospital could introduce selection bias; future studies should include multiple healthcare settings. Third, the use of city-level exposure metrics may lead to exposure misclassification; incorporating individual mobility data could improve accuracy. Fourth, the exclusion of composite heat-humidity indices limits the assessment of humid-heat effects; future work could adopt indices such as wet-bulb globe temperature. Finally, unmeasured confounders like medication adherence and socioeconomic status may affect the results; integrating individual-level cohort data would help address residual confounding.

## 5 Conclusion

This study demonstrates that in Panzhihua City, high temperature and high humidity can reduce the overall risk of hospital admissions for older patients with MetS complicated by cerebral infarction. However, this protective effect exhibits heterogeneity across different subgroups. A protective association was observed for males exposed to high temperature and high humidity, whereas for females, exposure to low temperature was protective. In patients aged 60–75, protective effects were found for exposure to relatively high temperature, low humidity, and high humidity. Therefore, public health policy makers can take preventive measures in advance by establishing targeted meteorological health risk warning systems.

## Data Availability

The original contributions presented in the study are included in the article/supplementary material, further inquiries can be directed to the corresponding author/s.
