# Supplementary material for: Effects of temperature and humidity on hospitalizations for metabolic syndrome with cerebral infarction among older adults in Panzhihua: a distributed lag non-linear model analysis
Source: Front Public Health. 2026 Mar 3;14:1674020. doi: 10.3389/fpubh.2026.1674020 (PMC12994406; doi:10.3389/fpubh.2026.1674020)

*Supplementary Material*

# Effects of Temperature and Humidity on Hospitalizations for Metabolic Syndrome with Cerebral Infarction among Older Adults in Panzhihua: A Distributed Lag Non-Linear Model Analysis

**Bingli Chen[1], Chunyan Zhou[1], Xiaoyi Liu[1], Deyun Luo[1], Jinxin Mo[1], Shiyang Li[1], Qian Zhu[2*†], Li Yin[2*†]**

[1] Department of Geriatrics, Panzhihua Central Hospital ,617000 Sichuan, China

[2] Clinical Medical Research Center, Meteorological Medical Research Center, Panzhihua Central Hospital ,617000 Sichuan, China

*** Correspondence:**

Qian Zhu, Ph.D. E-mail: zhuqian028@outlook.com

Clinical Medical Research Center, Meteorological Medical Research Center, Panzhihua Central Hospital

No. 34 Yikang Street, East Panzhihua City, 617000 Sichuan, China

Fax: +0812-2238177

Li Yin, Ph.D. E-mail: pzhyinli202507@163.com

Clinical Medical Research Center, Meteorological Medical Research Center, Panzhihua Central Hospital

No. 34 Yikang Street, East Panzhihua City, 617000 Sichuan, China

Fax: +0812-2224265

[†]These authors contributed equally to this work and share senior authorship.

**Supplementary Table 1 Lag effects of temperature on hospital admission for MetS complicated with cerebral infarction in the older adult(s) after changing model parameters.**

| Variables | Temperature | Lag1 | Lag7 | Lag14 | Lag21 |
|---|---|---|---|---|---|
| **Confounding factor** | | | | | |
| No pollutants | 5th | 0.990(0.823~1.191) | 0.966(0.888~0.051) | 0.933(0.869~1.001) | 1.028(0.900~1.173) |
| | 95th | 0.978(0.845~1.133) | 1.029(0.958~1.105) | 0.962(0.906~1.022) | 0.908(0.814~1.012) |
| No rain, speed and pressure | 5th | 1.009(0.841~1.212) | 0.974(0.895~1.061) | 0.947(0.884~1.016) | 1.020(0.893~1.165) |
| | 95th | 0.960(0.827~1.114) | 1.038(0.966~1.115) | 0.967(0.910~1.027) | 0.918(0.824~1.023) |
| **Degree of freedom** | | | | | |
| 2 | 5th | 1.011(0.853~1.198) | 0.961(0.893~1.036) | 0.961(0.903~1.023) | 1.102(0.981~1.237) |
| | 95th | 1.011(0.880~1.162) | 1.052(0.988~1.121) | 0.984(0.933~1.038) | 0.948(0.860~1.045) |
| 3 | 5th | 0.973(0.806~1.175) | 0.970(0.891~1.057) | 0.935(0.871~1.004) | 1.024(0.896~1.170) |
| | 95th | 0.992(0.853~1.155) | 1.028(0.957~1.104) | 0.966(0.909~1.026) | 0.906(0.813~1.011) |
| 4 | 5th | 1.014(0.830~1.238) | 0.965(0.880~1.058) | 0.923(0.855~0.996) | 1.025(0.888~1.183) |
| | 95th | 0.967(0.828~1.130) | 1.036(0.961~1.117) | 0.963(0.903~1.028) | 0.903(0.806~1.011) |

**Supplementary Table 2 Analysis of second-order interaction effects between temperature and humidity using a generalized linear regression model with quantitative data.**

| **Coefficients** | **β** | **SE** | **t value** | ***P* value** |
|---|---|---|---|---|
| DAT | $8.764\times10^{-2}$ | 7.167 | 1.223 | 0.222 |
| RH | $2.460\times10^{-2}$ | 2.026 | 1.214 | 0.225 |
| DAT^2 | $-4.296\times10^{-4}$ | 1.350 | -0.318 | 0.750 |
| RH^2 | $-2.921\times10^{-5}$ | 1.272 | -0.230 | 0.818 |
| DAT: RH | $-8.164\times10^{-4}$ | 5.021 | -1.626 | 0.104 |

**Supplementary Table 3 Testing the temperature and humidity interaction effect by tertile-based stratified analysis.**

| Coefficients | Quantile ($P_{33.3}$ and $P_{66.7}$) | | | | Quantile ($P_{25}$ and $P_{75}$) | | | |
|---|---|---|---|---|---|---|---|---|
| | β | SE | t value | *P* value | β | SE | t value | *P* value |
| **DAT** | | | | | | | | |
| Low | 1.985 | 3.112 | 0.638 | 0.524 | 2.757 | 4.519 | 0.610 | 0.542 |
| Medium | 5.082 | 10.795 | 0.471 | 0.638 | 7.166 | 14.205 | 0.504 | 0.614 |
| High | 2.208 | 2.423 | 0.911 | 0.362 | 2.563 | 2.841 | 0.902 | 0.367 |
| **RH** | | | | | | | | |
| Low | -1.307 | 3.051 | -0.428 | 0.669 | -1.462 | 4.313 | -0.339 | 0.735 |
| Medium | 9.428 | 11.535 | 0.817 | 0.414 | 12.798 | 15.402 | 0.831 | 0.406 |
| High | 7.811 | 4.232 | 1.846 | 0.065 | 8.334 | 4.960 | 1.680 | 0.093 |
| **DAT: RH** | | | | | | | | |
| Low: Low | 0.337 | 1.748 | 0.193 | 0.847 | 0.526 | 2.712 | 0.194 | 0.846 |
| Medium: Low | 3.706 | 6.273 | 0.591 | 0.555 | 4.016 | 8.724 | 0.460 | 0.645 |
| High: Low | 0.568 | 1.762 | 0.323 | 0.747 | 0.409 | 2.051 | 0.199 | 0.842 |
| Low: Medium | -5.920 | 6.622 | -0.894 | 0.371 | -8.388 | 9.624 | -0.872 | 0.384 |
| Medium: Medium | -19.406 | 23.753 | -0.817 | 0.414 | -26.104 | 31.429 | -0.831 | 0.406 |

| | | | | | | | | |
|---|---|---|---|---|---|---|---|---|
| High: Medium | -7.338 | 6.854 | -1.071 | 0.285 | -8.176 | 7.692 | -1.063 | 0.288 |
| Low: High | -3.957 | 2.167 | -1.826 | 0.068 | -4.665 | 2.744 | -1.700 | 0.089 |
| Medium: High | -17.588 | 10.481 | -1.678 | 0.094 | -18.337 | 11.903 | -1.541 | 0.124 |
| High: High | -7.104 | 6.645 | -1.069 | 0.285 | -6.389 | 6.014 | -1.062 | 0.288 |

**Supplementary Table 4 Comparison with Epidemiological Studies in Different Regions**

| Study Region (Climate Type) | Key Climate Features | Study Population | Conclusion |
|---|---|---|---|
| Panzhihua, China<br>Dry-Hot Valley Climate | High annual temperature, intense sunshine, low humidity, large diurnal temperature range | Older patients with MetS and cerebral infarction | First comprehensive study in a dry-hot valley climate. Reveals protective effects of high temperature and high humidity. |
| Beijing, China (26)<br>Temperate Monsoon Climate | Cold winters, hot summers, distinct seasonal variation | Ischemic stroke patients | Demonstrates cold temperature as a major risk factor. Highlights role of climate adaptation in modifying temperature effects. |
| Wuhu, China (36)<br>Subtropical Humid Monsoon Climate | Hot and humid summers, concentrated rainfall | General population with MetS | Shows extreme heat and humidity as risk factors for MetS mortality. |
| Northern Portugal (41)<br>Temperate Oceanic Climate | Mild year-round, high humidity, moderate rainfall | General stroke population | Identifies high humidity as a stroke risk factor. Suggests humidity effects may reverse in different climate contexts (humid vs. dry-hot). |
| Yaoundé, Cameroon (43)<br>Tropical Rainforest Climate | Consistently hot and very humid year-round | Diabetic patients | Shows highest disease incidence during most humid months. Confirms high humidity as metabolic risk in truly humid climates. |
| Jilin, China (45)<br>Temperate Continental Climate | Cold winters, large temperature range | older adult(s) with cardiovascular disease | Also suggests potential protective effect of high humidity. Challenges simplistic views of humidity as universally harmful. |

**Supplementary Figure 1 Trend chart of monthly hospitalization of older patients with MetS complicated with cerebral infarction in Panzhihua City.**

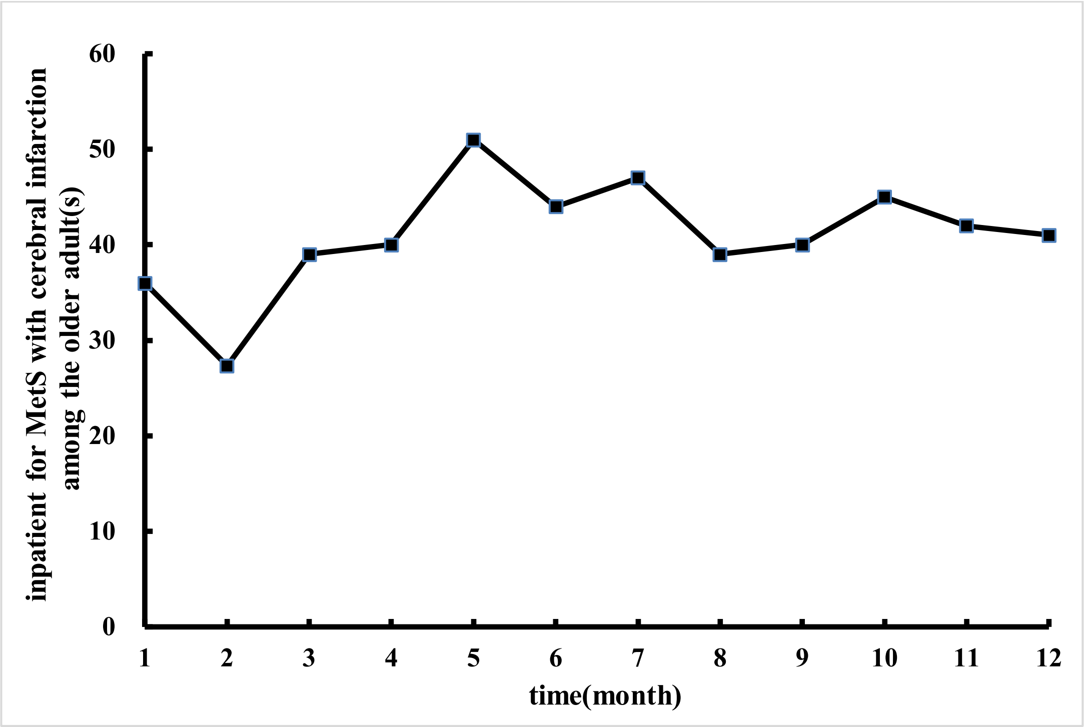

**Supplementary Figure 2 Trend of Monthly Average Temperature and Average RH in Panzhihua City from 2016 to 2020.**

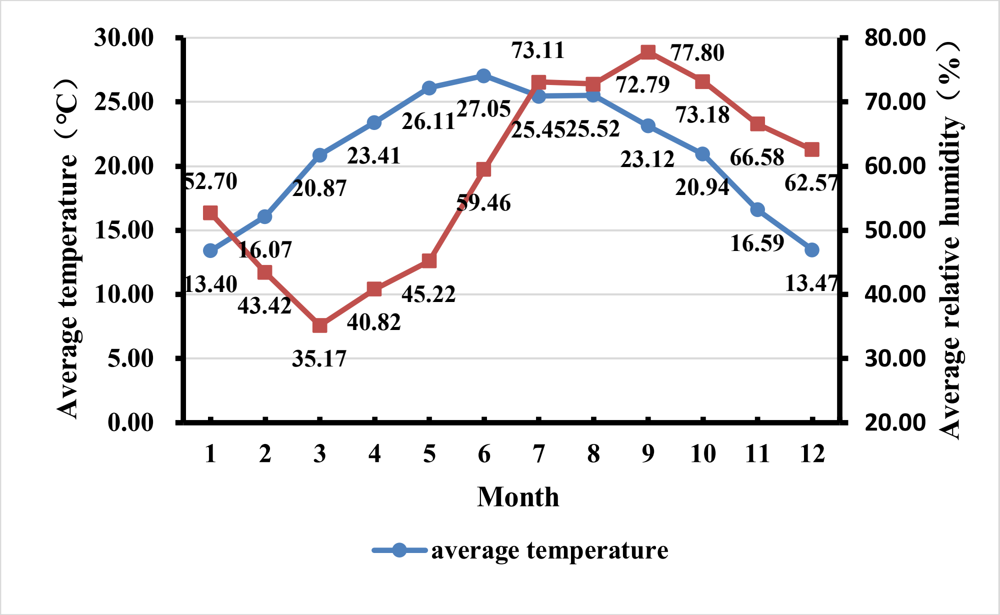

Supplement: Supplementary file 1 [file Data_Sheet_1.pdf]
